# Distribution and association of interpregnancy weight change with subsequent pregnancy outcomes in Asian women

**DOI:** 10.1038/s41598-023-31954-5

**Published:** 2023-03-24

**Authors:** Chee Wai Ku, Tuck Seng Cheng, Chee Onn Ku, Kathy Xinzhuo Zhou, Yin Bun Cheung, Keith M. Godfrey, Wee Meng Han, Fabian Yap, Jerry Kok Yen Chan, See Ling Loy

**Affiliations:** 1 Department of Reproductive Medicine, KK Women’s and Children’s Hospital, Singapore, 229899 Singapore; 2 Duke-NUS Medical School, Singapore, 169857 Singapore; 3 MRC Epidemiology Unit, Institute of Metabolic Science, University of Cambridge, Cambridge Biomedical Campus Box 285, Cambridge, CB2 0QQ UK; 4 Faculty of Science, National University of Singapore, Singapore, 119077 Singapore; 5 Lee Kong Chian School of Medicine, Nanyang Technological University, Singapore, 636921 Singapore; 6 Program in Health Services and Systems Research and Center for Quantitative Medicine, Duke-NUS Medical School, Singapore, 169857 Singapore; 7 Tampere Center for Child, Adolescent and Maternal Health Research, Tampere University, 33014 Tampere, Finland; 8 Medical Research Council Lifecourse Epidemiology Centre, University of Southampton, Southampton, SO16 6YD UK; 9 National Institute for Health Research Southampton Biomedical Research Centre, University of Southampton and University Hospital Southampton National Health Service Foundation Trust, Southampton, SO16 6YD UK; 10 Department of Dietetics, KK Women’s and Children’s Hospital, Singapore, 229899 Singapore; 11 Department of Paediatrics, KK Women’s and Children’s Hospital, Singapore, 229899 Singapore; 12 Yong Loo Lin School of Medicine, National University of Singapore, Singapore, 119077 Singapore

**Keywords:** Health policy, Nutrition, Weight management

## Abstract

The extent of interpregnancy weight change and its association with subsequent pregnancy outcomes among Asians remain unclear. We examined changes in maternal body mass index (BMI) between the first two deliveries and outcomes in the second delivery. Medical records of women with their first two consecutive deliveries between 2015 and 2020 at KK Women’s and Children’s Hospital, Singapore were retrieved. Gestational-age-adjusted BMI was determined by standardising to 12 weeks gestation and interpregnancy BMI change was calculated as the difference between both pregnancies. Pregnancy outcomes were analysed using modified Poisson regression models. Of 6264 included women with a median interpregnancy interval of 1.44 years, 40.7% had a stable BMI change within ± 1 kg/m^2^, 10.3% lost > 1 kg/m^2^, 34.3% gained 1–3 kg/m^2^ and 14.8% gained ≥ 3 kg/m^2^. Compared to women with stable BMI change, those with > 1 kg/m^2^ loss had higher risk of low birthweight (adjusted risk ratio [RR] 1.36; 95% confidence interval 1.02–1.80), while those with 1–3 kg/m^2^ gain had higher risks of large-for-gestational-age birth (1.16; 1.03–1.31), gestational diabetes (1.25; 1.06–1.49) and emergency Caesarean delivery (1.16; 1.03–1.31); these risks were higher in those with ≥ 3 kg/m^2^ gain. Our study strengthens the case for interpregnancy weight management to improve subsequent pregnancy outcomes.

## Introduction

The rates of overweight and obesity continue to increase worldwide^1^. In women, pregnancy is a life stage that can alter their weight trajectory due to the risk of weight gain during or between pregnancies^2,3^. Higher parity has been associated with higher pre-pregnancy body mass index (BMI) and subsequent development of obesity^4,5^. On average, women gain approximately 1 kg/m^2^ between consecutive pregnancies, with greater interpregnancy BMI gain observed in those with a higher BMI before pregnancy^6^.

In women who are overweight or obese, or underweight, the risks of adverse perinatal outcomes are well documented^7,8^. However, the extent to which interpregnancy weight change influences the risks of subsequent maternal and neonatal outcomes remain poorly understood^9^, and most studies have been focused on Western populations^10^. It is essential to personalize weight management planning for Asian women as they have higher health risks at lower BMI thresholds than Caucasian women^11^, and they have unique sociocultural factors which may influence weight management behaviours before, during, and after pregnancy^10^.

The interpregnancy period represents a unique phase of the reproductive life-course. A recent systematic review and meta-analysis with data pooled from 11 Western countries showed that women with interpregnancy weight gain had increased risks of gestational diabetes, hypertensive disorders, large-for-gestational-age birth, and Caesarean delivery, while those with interpregnancy weight loss had increased risks for preterm delivery and small-for-gestational-age birth^10^. In the present study, our aims were to (i) describe the distribution of weight changes in BMI between first and second pregnancies among Singaporean women and (ii) examine whether similar associations between interpregnancy BMI changes and pregnancy outcomes would be observed in Asian women, compared to those reported in the aforementioned meta-analysis among Caucasians^10^.

## Material and methods

Secondary routine healthcare data was retrieved from women with their first two consecutive deliveries from January 2015 to September 2020 at the KK Women’s and Children’s Hospital (KKH), Singapore. KKH houses the largest public maternity unit in Singapore and manages one-third of all live births in this country with approximately 12,000 deliveries every year, across a wide sociodemographic spectrum. We retrospectively extracted electronic medical records of women who had singleton births at ≥ 24 weeks gestation in the first and second pregnancies. Only women aged ≥ 21 years and conceived naturally in the first and second pregnancies were included. Women with missing information about BMI (at first and/ or second pregnancies) and interpregnancy interval were excluded. Ethics approval was obtained from the Centralised Institutional Review Board of SingHealth (reference 2020/2018). Informed consent was waived due to the retrospective nature of the study by the Centralised Institutional Review Board of SingHealth. All methods were performed in accordance with the relevant guidelines and regulations.

### Interpregnancy BMI change and interval

Maternal weight in kilograms and height in centimetres were routinely measured at the first antenatal appointment of the first and second deliveries. BMI, calculated as weight (in kilograms) divided by height (in metres) squared, at the first antenatal visit during the first and second pregnancies, was used to determine the interpregnancy BMI change. Given that gestational age at the first antenatal visit varied, the BMI measures were standardised separately in the first and second deliveries, by using linear regression with BMI at the first antenatal visit as dependent variable and gestational age centred at 12 weeks (i.e. the mean gestational age at the first antenatal visits where we had more data available to ensure a more accurate prediction of BMI) as the independent variable, calculating the residuals, and adding the residual values to the regression predicted mean BMI at 12 weeks. This is in keeping with data showing that weight in early pregnancy is a valid method for estimating pre-pregnancy weight^12^. We repeated the analysis by adjusting for maternal age and ethnicity in the multivariable linear regression model. Since a strong correlation (r > 0.95) was noted between both versions of predicted BMI at 12 weeks, the one that was derived using the simpler method without adjustment was used for all study analyses. The difference between gestational-age-adjusted BMI at both visits was then calculated as the change in BMI from the first to second deliveries and further categorized as BMI stable − 1 to < 1 kg/m^2^, BMI loss > 1 kg/m^2^, moderate BMI gain 1 to < 3 kg/m^2^ and excess BMI gain ≥ 3 kg/m^2^. The gestational-age-adjusted BMI at 12 weeks was used to represent the pre-pregnancy BMI in both pregnancies and was categorized using cut-offs for Asian populations: underweight (< 18.5 kg/m^2^), normal weight (18.5–22.9 kg/m^2^), overweight (23–27.49 kg/m^2^) and obese (≥ 27.5 kg/m^2^)^11^. The interpregnancy interval was calculated based on the period between the first delivery date and the second delivery conception date, which was derived by subtracting gestational age at delivery for the second birth from the duration between delivery dates of two consecutive births^13^.

### Pregnancy outcomes

Neonatal outcomes included preterm delivery (< 37 completed gestation weeks), low birthweight (< 2.5 kg), high birthweight (≥ 4 kg), small-for-gestational-age (SGA) and large-for-gestational-age (LGA). SGA and LGA were defined as birthweight for sex and gestational age below the 10th centile and above the 90th centile, respectively, using the algorithm reported by Mikolaiczyk et al.^14^ based on a reference sample of healthy livebirths from the Growing Up in Singapore Towards healthy Outcomes (GUSTO) cohort, which is the largest pregnancy cohort study involving approximately 1000 mother–child pairs in Singapore^15^. Maternal outcomes included gestational diabetes mellitus (GDM) as diagnosed by a risk-based, 2-point oral glucose tolerance test (OGTT) between 2015 and 2017^16^, and a universal 3-point OGTT between 2018 and 2020^17^, elective and emergency Caesarean deliveries. Gestational hypertensive disorders were not included in the analysis due to incomplete information recorded in the electronic medical database.

### Statistical analysis

The differences in characteristics between excluded and included women were compared using chi-square tests for categorical variables and independent t-tests for continuous variables. The associations of interpregnancy BMI change with subsequent pregnancy outcomes in the second pregnancy were examined using modified Poisson regression models to estimate risk ratios (RRs) and 95% confidence intervals (CIs)^18^. The change in interpregnancy BMI was included as a categorical exposure (BMI stable, loss, moderate gain, or excessive gain), with stable BMI used as the reference group, as conventionally used in other studies^10^. The models were adjusted for maternal age (continuous), ethnicity (categorical), gestational-age-adjusted BMI at 12 weeks in the first pregnancy (continuous), interpregnancy interval (continuous) and pregnancy outcomes in the first pregnancy (categorical).

As the effect of interpregnancy change on pregnancy outcomes may differ by maternal BMI at the beginning of the first pregnancy, we performed post-hoc analysis to examine whether there was any effect modification by weight status < 23 versus ≥ 23 kg/m^2^ at 12 weeks during the first pregnancy on any observed association. These models included categorical interpregnancy BMI change, weight status (effect modifier), the interaction terms between categorical interpregnancy BMI change weight status (3 degrees of freedom), and potential confounders as the independent variables. The results were stratified by weight status.

Sensitivity analyses were performed using a similar modified Poisson regression to analyse the associations of the crude (unstandardised for gestational age) change in interpregnancy BMI with pregnancy outcomes, with confounders adjustment. These analyses were restricted to those with measures before or at 12 weeks gestation for both pregnancies. Statistical analyses were performed using Stata 16 (Stata, College Station, TX, USA).

## Results

### Women’s characteristics

This study initially enrolled 7095 women with singleton first and second pregnancies. Of these, we excluded 831 women without BMI measured in one of the pregnancies (n = 772) or in both pregnancies (n = 59), leaving 6264 women in the final sample. Compared to excluded women, the included women tended to be older by only 0.5 years on average (28.4 vs. 27.9 years, *p* = 0.015) (see Supplementary Table S1 online). All other background variables were similar between the women included and excluded from the analysis (each *p* > 0.05).

Of 6264 included women, 40.7% had a stable interpregnancy BMI (− 1 to < 1 kg/m^2^), 10.3% had BMI loss (> 1 kg/m^2^), 34.3% had moderate BMI gain (1 to < 3 kg/m^2^) and 14.8% had excess BMI gain (≥ 3 kg/m^2^) (Table 1). Women of younger age, Malay ethnicity and with higher BMI in the first pregnancy tended to experience excess BMI gain between their first two pregnancies.

**Table 1:** Characteristics of participants according to their interpregnancy BMI change status (n = 6264).

Characteristics	Total	BMI change status between first two pregnancies			
		Stable	Loss	Moderate gain	Excess gain
		(− 1 to < 1 kg/m^2^)	(> 1 kg/m^2^)	(1 to < 3 kg/m^2^)	(≥ 3 kg/m^2^)
	n = 6264	n = 2548; 40.7%	n = 643; 10.3%	n = 2146; 34.3%	n = 927; 14.8%
Maternal age in the first pregnancy, years	28.36 ± 4.31	28.58 ± 4.32	28.61 ± 4.64	28.49 ± 4.17	27.27 ± 4.18
Ethnicity					
Chinese	2600 (41.5)	1192 (46.8)	275 (42.8)	916 (42.7)	217 (23.4)
Malay	1902 (30.4)	704 (27.6)	182 (28.3)	620 (28.9)	396 (42.7)
Indian	666 (10.6)	231 (9.1)	79 (12.3)	233 (10.9)	123 (13.3)
Others	1096 (17.5)	421 (16.5)	107 (16.6)	377 (17.6)	191 (20.6)
BMI at 12-week gestation in the first pregnancy, kg/m^2^	23.76 ± 4.97	23.19 ± 5.04	25.36 ± 5.44	23.35 ± 4.48	25.17 ± 5.02
BMI categories at 12-week gestation in the first pregnancy					
Underweight (< 18.5 kg/m^2^)	585 (9.3)	319 (12.6)	17 (2.7)	197 (9.2)	52 (5.6)
Normal weight (18.5–22.9 kg/m^2^)	2719 (43.4)	1209 (47.4)	242 (37.6)	974 (45.4)	294 (31.7)
Overweight (23–27.4 kg/m^2^)	1785 (28.5)	607 (23.8)	208 (32.3)	656 (30.6)	314 (33.9)
Obesity (≥ 27.5 kg/m^2^)	1175 (18.8)	413 (16.2)	176 (27.4)	319 (14.8)	267 (28.8)
BMI at 12-week gestation in the second pregnancy, kg/m^2^	24.92 ± 5.40	23.33 ± 5.03	23.35 ± 5.27	25.23 ± 4.58	29.65 ± 5.32
BMI categories at 12-week gestation in the second pregnancy					
Underweight (< 18.5 kg/m^2^)	416 (6.6)	286 (11.2)	81 (12.6)	48 (2.2)	1 (0.1)
Normal weight (18.5–22.9 kg/m^2^)	2262 (36.1)	1210 (47.5)	302 (47.0)	694 (32.3)	56 (6.0)
Overweight (23–27.4 kg/m^2^)	1972 (31.5)	626 (24.6)	145 (22.6)	887 (41.3)	314 (33.9)
Obesity (≥ 27.5 kg/m^2^)	1614 (25.8)	426 (16.7)	115 (17.8)	517 (24.2)	556 (60.0)
Interpregnancy BMI change, kg/m^2^	0.97 (− 0.04 to 2.21)	0.19 (− 0.25 to 0.59)	− 1.72 (− 2.40 to -1.31)	1.84 (1.40–2.32)	4.02 (3.45–4.95)
Interpregnancy interval, years	1.44 (0.89–2.19)	1.39 (0.88–2.04)	1.34 (0.84–1.97)	1.49 (0.89–2.27)	1.61 (0.97–2.61)

Data are presented as number (percentage) for categorical variables, and as mean ± standard deviation or median (25th–75th percentiles) for continuous variables. BMI, body mass index.

### Distribution of interpregnancy BMI change

**Figure 1:**
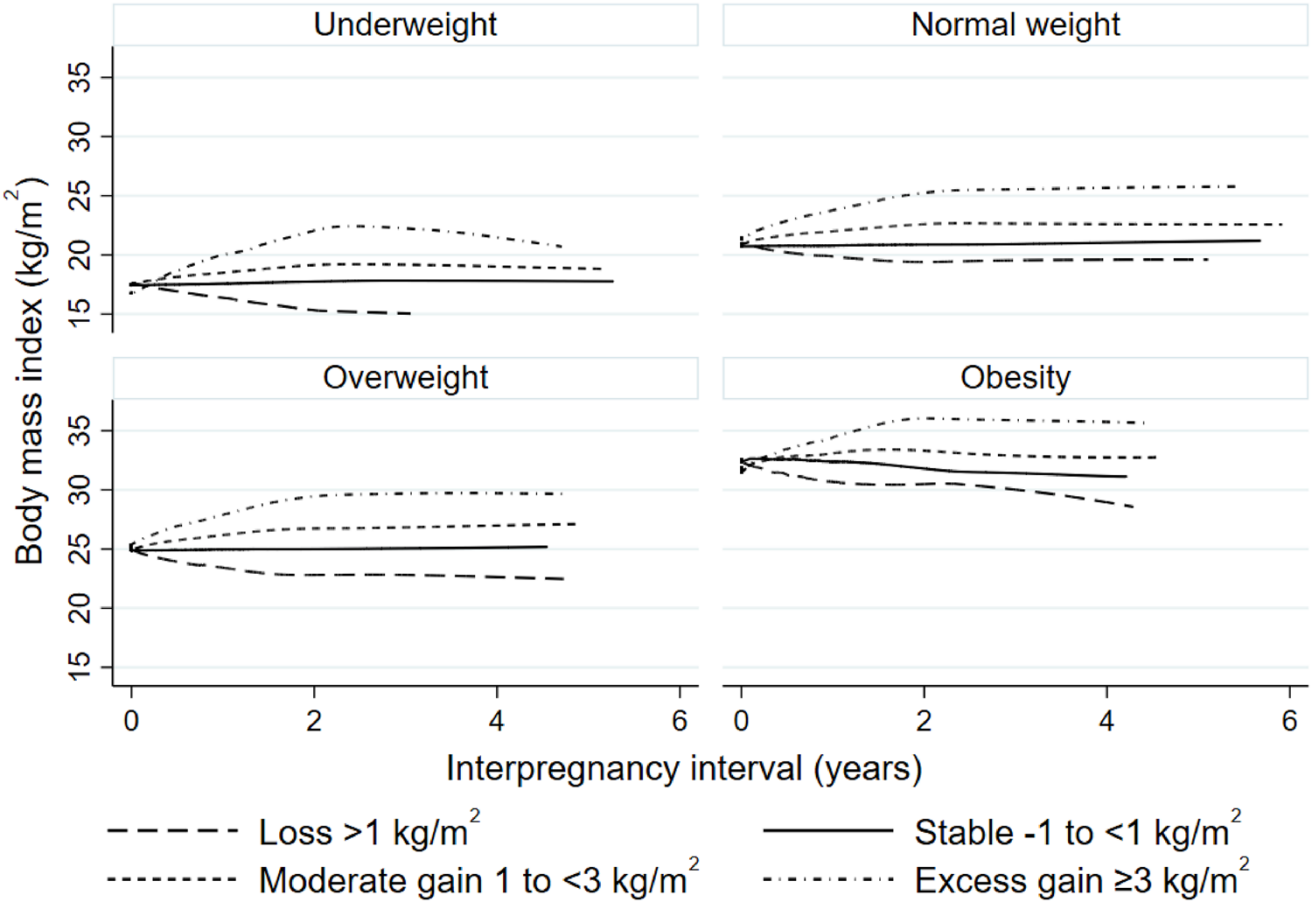
Cross-sectional trends of BMI change over interpregnancy interval. BMI categories of women were measured at 12-week gestation in the first pregnancy (n = 6264). BMI, body mass index.

**Figure 2:**
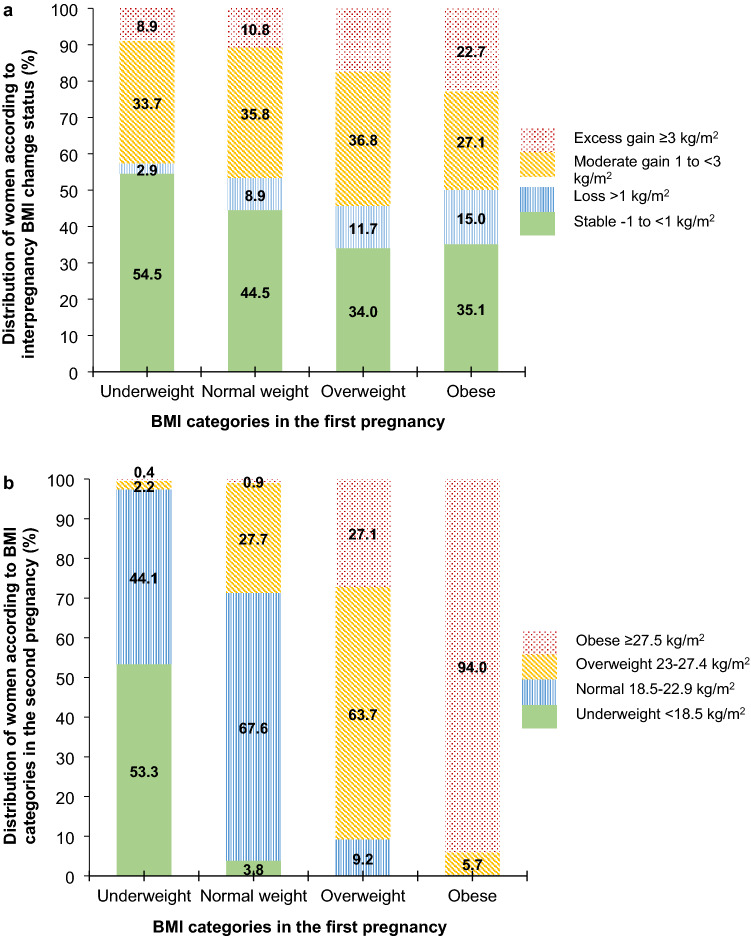
Interpregnancy BMI change status and BMI categories in the second pregnancy by BMI categories in the first pregnancy. Bar chart showing the distribution of **(a)** body mass index (BMI) change status between first two pregnancies and **(b)** BMI categories at 12-week gestation in the second pregnancy, by BMI categories of women at 12-week gestation in the first pregnancy (n = 6264). BMI categories were classified based on the cut-offs for Asian populations. BMI, body mass index.

Overall, BMI tended to change (increase or decrease) among women who gave the second birth in the first two years after the first delivery and was stable at that level among women who gave the second birth later, regardless of the initial weight status (Fig. 1). Women who were overweight and obese in their first pregnancy tended to experience interpregnancy BMI loss or gain as compared to those who were underweight and normal weight, who tended to be BMI stable (*p* < 0.001) (Fig. 2a). In particular, those women who were overweight or obese had higher BMI loss than women with a normal BMI (median BMI loss 1.9 vs. 1.5 kg/m2, *p* < 0.001) (see Supplemental Fig. S1 online).

In total, 24.5% of women gained weight between pregnancies and progressed to a higher BMI category; while 5.4% of women lost weight and dropped to a lower BMI category. Although at least 90% of women who were overweight or obese in the first pregnancy remained at least overweight in the second pregnancy, nearly two-thirds of women with normal weight and half of women who were underweight remained in the same weight status in the first and second pregnancies (Fig. 2b). Similar distributions of interpregnancy BMI change status and weight status in the second pregnancy were observed across weight status in the first pregnancy based on the WHO conventional cut-offs (see Supplementary Table S2 online).

### Interpregnancy BMI change and subsequent pregnancy outcomes

Compared to women with a stable BMI from the first to the second pregnancy, those with BMI loss had a higher risk of low birthweight delivery (RR 1.36; 95% CI 1.02–1.80). Women with moderate BMI gain had higher risks of LGA birth (1.16; 1.03–1.31), GDM (1.25; 1.06–1.49) and emergency Caesarean delivery (1.16; 1.03–1.31) in the second pregnancy; these risks were higher in those with excess BMI gain (Table 2). Similar findings were obtained in a sensitivity analysis using crude interpregnancy BMI change (see Supplementary Table S3 online). In women with BMI ≥ 23 kg/m^2^, BMI loss was associated with increased risk of low birthweight (1.64; 1.09–2.47) and SGA deliveries (1.54; 1.02–2.34) (Table 3). In women with BMI < 23 kg/m^2^, moderate (1.31; 1.07–1.59) and excess BMI gains (1.35; 1.04–1.77) were associated with an increased risk of emergency Caesarean.

**Table 2:** Association between interpregnancy BMI change status and outcomes of second pregnancy.

Outcomes of second pregnancy	BMI change status between first two pregnancies						
	Stable	Loss		Moderate gain		Excess gain	
	(-1 to < 1 kg/m^2^)	(> 1 kg/m^2^)		(1 to < 3 kg/m^2^)		(≥ 3 kg/m^2^)	
	n (%)	n (%)	RR (95% CI)	n (%)	RR (95% CI)	n (%)	RR (95% CI)
Offspring birth weight							
Normal 2.5 to < 4 kg	2341 (92.2)	574 (89.7)	1.00	1965 (92.0)	1.00	839 (90.8)	1.00
Low < 2.5 kg	163 (6.4)	55 (8.6)	1.36 (1.02, 1.80)	136 (6.4)	1.00 (0.81, 1.25)	57 (6.2)	0.97 (0.72, 1.31)
High ≥ 4 kg	35 (1.4)	11 (1.7)	0.88 (0.46, 1.69)	34 (1.6)	1.09 (0.68, 1.74)	28 (3.0)	1.62 (0.97, 2.71)
Offspring birth size							
AGA 10–90 percentile	1932 (76.2)	488 (76.2)	1.00	1583 (74.1)	1.00	643 (69.7)	1.00
SGA < 10 percentile	206 (8.1)	54 (8.4)	1.15 (0.87, 1.52)	161 (7.5)	0.94 (0.78, 1.14)	61 (6.6)	0.84 (0.64, 1.11)
LGA > 90 percentile	399 (15.7)	98 (15.3)	0.87 (0.72, 1.05)	391 (18.3)	1.16 (1.03, 1.31)	219 (23.7)	1.40 (1.21, 1.61)
Preterm delivery < 37 weeks							
No	2385 (93.6)	592 (92.1)	1.00	2012 (93.8)	1.00	863 (93.1)	1.00
Yes	163 (6.4)	51 (7.9)	1.08 (0.80, 1.45)	134 (6.2)	0.96 (0.78, 1.20)	64 (6.9)	1.07 (0.81, 1.41)
Gestational diabetes							
No	2352 (92.3)	580 (90.2)	1.00	1956 (91.1)	1.00	806 (86.9)	1.00
Yes	196 (7.7)	63 (9.8)	1.04 (0.81, 1.34)	190 (8.9)	1.25 (1.06, 1.49)	121 (13.1)	1.63 (1.35, 1.97)
Mode of delivery							
Vaginal delivery	1892 (76.7)	541 (74.4)	1.00	1573 (74.5)	1.00	718 (74.9)	1.00
Elective caesarean	345 (14.0)	102 (14.0)	1.01 (0.89, 1.15)	301 (14.3)	1.02 (0.95, 1.10)	123 (12.8)	1.04 (0.95, 1.15)
Emergency caesarean	229 (9.3)	84 (11.6)	1.09 (0.93, 1.28)	238 (11.3)	1.16 (1.03, 1.31)	118 (12.3)	1.14 (1.00, 1.32)

Risk ratios are adjusted for maternal age and BMI at 12-week gestation in the first pregnancy, ethnicity, interpregnancy interval and respective pregnancy outcomes in the first pregnancy. BMI stable (− 1 to < 1 kg/m^2^) serves as the reference group. BMI, body mass index; RR, risk ratio; CI, confidence interval; AGA, appropriate for gestational age; SGA, small-for-gestational-age; LGA, large-for-gestational-age.

**Table 3:** Association between interpregnancy BMI change status and outcomes of second pregnancy, by weight status at 12-week gestation in the first pregnancy.

Outcomes of second pregnancy	BMI < 23 kg/m^2^			BMI ≥ 23 kg/m^2^			P-interaction
	Loss	Moderate gain	Excess gain	Loss	Moderate gain	Excess gain	
	(> 1 kg/m^2^)	(1 to < 3 kg/m^2^)	(≥ 3 kg/m^2^)	(> 1 kg/m^2^)	(1 to < 3 kg/m^2^)	(≥ 3 kg/m^2^)	
	RR (95% CI)	RR (95% CI)	RR (95% CI)	RR (95% CI)	RR (95% CI)	RR (95% CI)	
Offspring birth weight							
Low < 2.5 kg (vs. Normal 2.5 to < 4 kg)	1.22 (0.79, 1.90)	0.84 (0.63, 1.11)	1.07 (0.72, 1.59)	1.64 (1.09, 2.47)	1.43 (0.99, 2.07)	1.06 (0.66 2.71)	0.076
High ≥ 4 kg (vs. Normal 2.5 to < 4 kg)	0.52 (0.07, 4.04)	1.30 (0.56, 3.02)	3.00 (1.17, 7.68)	0.93 (0.46, 1.86)	1.01 (0.58, 1.76)	1.34 (0.74, 2.43)	0.428
Offspring birth size							
SGA < 10 percentile (vs. AGA 10–90 percentile)	0.96 (0.63, 1.45)	0.84 (0.67, 1.06)	0.88 (0.62, 1.25)	1.54 (1.02, 2.34)	1.29 (0.91, 1.83)	0.90 (0.58, 1.42)	0.078
LGA > 90 percentile (vs. AGA 10–90 percentile)	0.73 (0.49, 1.09)	1.16 (0.96, 1.40)	1.67 (1.30, 2.15)	0.88 (0.71, 1.10)	1.13 (0.97, 1.31)	1.23 (1.03, 1.46)	0.092
Preterm delivery < 37 weeks							
Yes (vs. No)	1.03 (0.65, 1.65)	0.82 (0.60, 1.12)	1.24 (0.81, 1.88)	1.09 (0.74, 1.61)	1.13 (0.83, 1.53)	1.02 (0.71, 1.48)	0.327
Gestational diabetes							
Yes (vs. No)	1.22 (0.76, 1.95)	1.11 (0.83, 1.48)	1.77 (1.20, 2.61)	0.99 (0.74, 1.31)	1.27 (1.03, 1.58)	1.50 (1.20, 1.88)	0.541
Mode of delivery							
Elective caesarean (vs. vaginal delivery)	1.10 (0.89, 1.36)	1.09 (0.97, 1.22)	1.09 (0.92, 1.28)	0.97 (0.84, 1.13)	0.97 (0.88, 1.07)	1.02 (0.91 1.16)	0.348
Emergency caesarean (vs. vaginal delivery)	1.34 (0.96, 1.88)	1.31 (1.07, 1.59)	1.35 (1.04, 1.77)	0.98 (0.82, 1.17)	1.06 (0.91, 1.24)	1.05 (0.89, 1.23)	0.133

Risk ratios are adjusted for maternal age and BMI at 12-week gestation in the first pregnancy, ethnicity, interpregnancy interval and respective pregnancy outcomes in the first pregnancy. BMI stable (− 1 to < 1 kg/m^2^) serves as the reference group. BMI, body mass index; RR, risk ratio; CI, confidence interval; AGA, appropriate for gestational age; SGA, small-for-gestational-age; LGA, large-for-gestational-age.

## Discussion

In this cohort that included 6264 women, about a quarter increased while 5% lowered their BMI category between their first and second pregnancies. Approximately half gained ≥ 1 kg/m^2^, of which one-third had excess gain of ≥ 3 kg/m^2^; only 10% lost > 1 kg/m^2^ between pregnancies. Overall, BMI tended to change among women who birthed their second child in the first two years after the first delivery, and was stable among women who birthed their second child later, regardless of the initial weight status. Interpregnancy BMI gain was associated with increased risks of LGA, GDM and emergency Caesarean delivery in the second pregnancy. Conversely, an increased risk of low birthweight was observed in women with BMI loss between their first two pregnancies. When the results were further stratified by BMI in the first pregnancy, a higher risk of emergency Caesarean delivery was evident in women with a BMI < 23 kg/m^2^ experiencing interpregnancy BMI gain, while higher risks of low birthweight and SGA were evident in women with a BMI ≥ 23 kg/m^2^ experiencing interpregnancy BMI loss.

The interpregnancy period is a valuable opportunity to address pregnancy complications and optimise health for the next pregnancy and the rest of the life-course. Despite recommendations to return to pre-pregnancy weight between 6 and 12 months postpartum, with the goal of a normal BMI^19^, about half the women in our study increased their BMI during the first two years post-delivery instead. A study conducted among Caucasian women also showed similar findings, where almost 20% of normal-weight women became overweight or obese in their next pregnancy, whereas more than 90% of overweight or obese women maintained their status in the next pregnancy^20^. This highlights the urgent need to implement intervention strategies that include targeted lifestyle modifications to prevent increased BMI during the interpregnancy period.

Interpregnancy BMI gain and the associated increased risks of subsequent LGA, GDM and emergency Caesarean delivery are consistent with previous studies^9,10,21^. These adverse complications could be the result of reduced insulin sensitivity due to interpregnancy weight gain accompanied by body fat rather than muscle gain, which is common among Asians^20,22–25^. The increased risk of emergency Caesarean delivery in women with an initial BMI < 23 kg/m^2^ is consistent with a recent meta-analysis^23^, suggesting an increased susceptibility of lean women to subsequent delivery complications in response to weight gain between pregnancies. However, the indications for emergency Caesarean delivery were unclear in our data and should be further examined in future studies. Similarly, interpregnancy BMI gain has been associated with increased risks of hypertensive disorders^9,26^ and stillbirth^10^, but we were unable to analyse these outcomes due to incomplete outcome data. In view of multiple adverse pregnancy outcomes, long-term obesity, and related health risks in women and their offspring, our study, together with many others^13,27–32^, call for nationwide efforts to break the vicious cycle of interpregnancy weight gain and poor metabolic health.

We found that offspring of women with BMI loss between their first two pregnancies had a higher risk of low birthweight. This is supported by a study on interpregnancy weight change among women in three consecutive pregnancies, showing that BMI loss was associated with an increased risk of low placental weight and SGA births^33^. Another study also showed that a decrease in BMI > 1 kg/m^2^ between the first two consecutive births was associated with a higher risk of low birthweight (< 2.5 kg)^34^. This phenomenon could be explained by insulin sensitivity induced by weight loss, resulting in less glucose crossing the placenta, which contributed to an increased risk of small fetal size^23^. A meta-analysis showed that interpregnancy weight loss and SGA was only apparent in women with initial BMI < 25 kg/m^2^, but not among those with BMI ≥ 25 kg/m^2^^10^. However, our study observed that women with BMI ≥ 23 kg/m^2^ in the first pregnancy who lost weight during the interpregnancy interval had a higher risk of low birthweight and SGA. Although not reaching statistical significance, women of BMI < 23 kg/m^2^ in the first pregnancy with an interpregnancy BMI loss also had a higher risk of low birthweight, albeit with a smaller effect size. These findings should be interpreted with caution as they may be attributed to the greater weight loss among women who were overweight or obese within the interpregnancy interval of 1–2 years, compared with women with a normal BMI (BMI loss 1.9 vs. 1.5 kg/m^2^, *p* < 0.001) (see Supplemental Fig. S1 online). In addition, unlike other studies that showed a reduction in the risk of adverse pregnancy outcomes among overweight and obese women who lost weight^10,20,21,23^, our study did not find a significant reduction in risk among women with BMI ≥ 23 kg/m^2^ who lost weight. Despite the current emphasis on BMI, it represents a crude measure of adiposity and an imperfect assessment of metabolic health^35^. This was highlighted by a recent study that showed that metabolic health status, rather than BMI, played a greater role in fecundability^36^. Therefore, interpregnancy BMI loss may not truly reflect the metabolic health status of our study participants, which confounds the positive effects of weight loss in overweight and obese women. Furthermore, changes in body composition and fat distribution between pregnancies, and gestational weight gain (GWG) during pregnancy in overweight or obese women can impact subsequent pregnancy outcomes^37^. The lack of metabolic health, GWG and other data in our study precludes making recommendations for the amount of weight loss to improve pregnancy outcomes, and further studies including this information are needed to make such recommendations.

Despite the higher risk of low birthweight and SGA in women with BMI ≥ 23 kg/m^2^ who lost weight during the interpregnancy interval, it is important to balance this with the benefits of achieving a normal BMI, especially in women living with obesity, given the potential for other adverse perinatal outcomes, such as GDM, hypertensive disorders of pregnancy, macrosomia, birth trauma, and stillbirth^7,8^. Based on the trend of interpregnancy BMI change, the first two years post-delivery likely represents the best window of opportunity to intervene to return to pre-pregnancy BMI, regardless of initial weight status. Effective lifestyle interventions that aim to limit postpartum weight retention during this window are crucial to improving perinatal outcomes. Such interventions should ideally be engaging, grounded by behaviour change theories, and integrate components of both diet and physical activity^38^. An electronic health intervention for postpartum women with excessive GWG resulted in restrained eating, along with decreased uncontrolled eating and energy intake^39^. However, other behaviours such as emotional eating, physical activity, and sedentary time remain unchanged^39^. To improve the success of lifestyle interventions, it is essential to identify additional facilitators and barriers faced by these women. Although these were identified among overweight and obese women trying to conceive^40^, it remains unclear whether such findings are applicable to women of normal weight.

This is the first study to investigate the distribution and outcomes of interpregnancy weight change in Asian women, with a substantial sample size of women from the three largest ethnicities in Singapore (Chinese, Malay, and Indian) where the findings may be generalizable to other Asian populations. However, the study employed statistical modelling to predict the maternal BMI at 12 weeks and used it as the pre-pregnancy BMI. This might result in misclassification of weight status and interpregnancy weight change categories. In addition, since BMI is an imperfect measurement of metabolic health^35^, future studies should investigate how other markers of metabolic health, such as insulin resistance, lipid profile and body composition, are associated with adverse perinatal outcomes. The GDM screening policy underwent a transition during the study period, from a risk-based 2-point OGTT between 2015 and 2017 to a universal 3-point OGTT from 2018 to 2020, thus, the incidence of GDM may be underestimated in the earlier years^41,42^. We did not account for the association of GWG with adverse perinatal outcomes, including fetal growth, preterm delivery, GDM, and Caesarean delivery^37^. Hence, we are unable to determine whether the association of interpregnancy BMI with adverse perinatal outcomes would be mediated by GWG, which should be a focus for future studies. We did not evaluate other adverse pregnancy outcomes such as intervening miscarriage, as ascertainment of this outcome is known to be incomplete, while our study was underpowered to examine low prevalence outcomes such as stillbirth. We did not account for the socioeconomic status and lifestyle habits of the women in the analysis due to the lack of data from medical records. Finally, long-term outcomes of these women and their offspring were not available to provide insights on their long-term health.

## Conclusion

This study has shown that a large proportion of women increase their BMI, and a small proportion decrease their BMI between their first two pregnancies. An increase and a decrease in BMI between pregnancies are associated with a higher risk of adverse outcomes in the second pregnancy. These findings highlight the importance of interpregnancy weight management to achieve better pregnancy outcomes subsequently. However, the recommended magnitude of weight loss beyond their pre-pregnancy weight remains unclear, especially for those who are overweight or obese, where a loss > 1 kg/m^2^ was associated with SGA and low birthweight. Future studies should examine the role of interpregnancy weight management interventions among Asian women, and to examine the role of metabolic health in adverse pregnancy outcomes with measurement of GWG, body composition and metabolic biomarkers. This will shed light on possible aetiologies of low birthweight/SGA and weight loss and guide personalized interventions and BMI targets for women with lean BMI and those who are overweight or obese.

## Supplementary Information

Supplementary Information.

## Data Availability

Please contact the corresponding author for more information.

## References

[1] NCD Risk Factor Collaboration (NCD-RisC) (2017). Worldwide trends in body-mass index, underweight, overweight, and obesity from 1975 to 2016: a pooled analysis of 2416 population-based measurement studies in 128·9 million children, adolescents, and adults. Lancet.

[2] Gunderson EP (2009). Childbearing and obesity in women: Weight before, during, and after pregnancy. Obstet. Gynecol. Clin. North Am..

[3] Nehring I, Schmoll S, Beyerlein A, Hauner H, von Kries R (2011). Gestational weight gain and long-term postpartum weight retention: A meta-analysis. Am. J. Clin. Nutr..

[4] Bastian LA, West NA, Corcoran C, Munger RG (2005). Number of children and the risk of obesity in older women. Prev. Med..

[5] Hill B (2017). Is parity a risk factor for excessive weight gain during pregnancy and postpartum weight retention? A systematic review and meta-analysis. Obes. Rev..

[6] Sumithran P (2018). How common is substantial weight gain after pregnancy?. Obes. Res. Clin. Pract..

[7] Dsouza R, Horyn I, Pavalagantharajah S, Zaffar N, Jacob CE (2019). Maternal body mass index and pregnancy outcomes: a systematic review and metaanalysis. Am. J. Obstet. Gynecol. MFM.

[8] Liu P (2016). Association between perinatal outcomes and maternal pre-pregnancy body mass index. Obes. Rev..

[9] Teulings N, Masconi KL, Ozanne SE, Aiken CE, Wood AM (2019). Effect of interpregnancy weight change on perinatal outcomes: Systematic review and meta-analysis. BMC Pregnancy Childbirth.

[10] Nagpal TS (2022). Does prepregnancy weight change have an effect on subsequent pregnancy health outcomes? A systematic review and meta-analysis. Obes. Rev..

[11] WHO Expert Consultation (2004). Appropriate body-mass index for Asian populations and its implications for policy and intervention strategies. Lancet.

[12] Inskip H (2021). Measured weight in early pregnancy is a valid method for estimating pre-pregnancy weight. J. Dev. Orig. Health Dis..

[13] Ziauddeen N (2022). Interpregnancy weight gain and childhood obesity: Analysis of a UK population-based cohort. Int. J. Obes. (Lond.).

[14] Mikolajczyk RT (2011). A global reference for fetal-weight and birthweight percentiles. Lancet.

[15] Soh SE (2014). Cohort profile: Growing up in Singapore towards healthy outcomes (GUSTO) birth cohort study. Int. J. Epidemiol..

[16] Alberti KG, Zimmet PZ (1998). Definition, diagnosis and classification of diabetes mellitus and its complications Part 1: Diagnosis and classification of diabetes mellitus provisional report of a WHO consultation. Diabet. Med..

[17] World Health Organization (2014). Diagnostic criteria and classification of hyperglycaemia first detected in pregnancy: A World Health Organization Guideline. Diabetes Res. Clin. Pract..

[18] Zou G (2004). A modified Poisson regression approach to prospective studies with binary data. Am. J. Epidemiol..

[19] Louis JM, Bryant A, Ramos D, Stuebe A, Blackwell SC (2019). Interpregnancy Care. Am. J. Obstet. Gynecol..

[20] McBain RD, Dekker GA, Clifton VL, Mol BW, Grzeskowiak LE (2016). Impact of inter-pregnancy BMI change on perinatal outcomes: A retrospective cohort study. Eur. J. Obstet. Gynecol. Reprod. Biol..

[21] Martínez-Hortelano JA (2021). Interpregnancy weight change and gestational diabetes mellitus: A systematic review and meta-analysis. Obesity (Silver Spring).

[22] Gunderson EP (2008). Childbearing may increase visceral adipose tissue independent of overall increase in body fat. Obesity (Silver Spring).

[23] Oteng-Ntim E (2018). Interpregnancy weight change and adverse pregnancy outcomes: A systematic review and meta-analysis. BMJ Open.

[24] Retnakaran R, Hanley AJ, Connelly PW, Sermer M, Zinman B (2006). Ethnicity modifies the effect of obesity on insulin resistance in pregnancy: A comparison of Asian, South Asian, and Caucasian women. J. Clin. Endocrinol. Metab..

[25] Sorbye LM, Skjaerven R, Klungsoyr K, Morken NH (2017). Gestational diabetes mellitus and interpregnancy weight change: A population-based cohort study. PLoS Med..

[26] Kawakita T, Downs SK, Franco S, Ghofranian A, Thomas A (2022). Interpregnancy body mass index change and risk of hypertensive disorders in pregnancy. J. Matern. Fetal Neonatal Med..

[27] Adane AA, Dobson A, Tooth L, Mishra GD (2018). Maternal preconception weight trajectories are associated with offsprings' childhood obesity. Int. J. Obes. (Lond.).

[28] Alwan NA, Grove G, Taylor E, Ziauddeen N (2020). Maternal weight change between successive pregnancies: An opportunity for lifecourse obesity prevention. Proc. Nutr. Soc..

[29] Catalano PM, Ehrenberg HM (2006). The short- and long-term implications of maternal obesity on the mother and her offspring. BJOG.

[30] Derraik JG, Ayyavoo A, Hofman PL, Biggs JB, Cutfield WS (2015). Increasing maternal prepregnancy body mass index is associated with reduced insulin sensitivity and increased blood pressure in their children. Clin. Endocrinol. (Oxf.).

[31] Endres LK (2015). Postpartum weight retention risk factors and relationship to obesity at 1 year. Obstet. Gynecol..

[32] Wahabi HA, Fayed AA, Tharkar S, Esmaeil SA, Bakhsh H (2019). Postpartum weight retention and cardiometabolic risk among Saudi women: A follow-up study of RAHMA subcohort. Biomed. Res. Int..

[33] Wallace JM, Bhattacharya S, Horgan GW (2017). Weight change across the start of three consecutive pregnancies and the risk of maternal morbidity and SGA birth at the second and third pregnancy. PLoS One.

[34] Bogaerts A (2013). Interpregnancy weight change and risk for adverse perinatal outcome. Obstet. Gynecol..

[35] Cirulli ET (2019). Profound perturbation of the metabolome in obesity is associated with health risk. Cell Metab..

[36] Loy SL (2022). Metabolic health status and fecundability in a Singapore preconception cohort study. Am. J. Obstet. Gynecol..

[37] Champion ML, Harper LM (2020). Gestational weight gain: Update on outcomes and interventions. Curr. Diabetes Rep..

[38] van der Pligt P (2013). Systematic review of lifestyle interventions to limit postpartum weight retention: Implications for future opportunities to prevent maternal overweight and obesity following childbirth. Obes. Rev..

[39] Bijlholt M, Ameye L, Van Uytsel H, Devlieger R, Bogaerts A (2021). The INTER-ACT E-Health supported lifestyle intervention improves postpartum food intake and eating behavior, but not physical activity and sedentary behaviour—A randomized controlled trial. Nutrients.

[40] Ku CW (2022). Developing a lifestyle intervention program for overweight or obese preconception, pregnant and postpartum women using qualitative methods. Sci. Rep..

[41] Chi C (2018). Impact of adopting the 2013 World Health Organization criteria for diagnosis of gestational diabetes in a multi-ethnic Asian cohort: A prospective study. BMC Pregnancy Childbirth.

[42] Chong YS (2014). Ethnic differences translate to inadequacy of high-risk screening for gestational diabetes mellitus in an Asian population: A cohort study. BMC Pregnancy Childbirth.

