# Supplementary material for: Distribution and association of interpregnancy weight change with subsequent pregnancy outcomes in Asian women
Source: Sci Rep. 2023 Mar 24;13:4834. doi: 10.1038/s41598-023-31954-5 (PMC10039003; doi:10.1038/s41598-023-31954-5)
Supplement: Supplementary file 1 — Supplementary Information. [file 41598_2023_31954_MOESM1_ESM.docx]

**Supplementary Table S1. Characteristics of participants according to their inclusion status in the present study.**

| **Characteristics** | **Excluded (n=831)** | **Included (n=6264)** | **P value^a^** |
| --- | --- | --- | --- |
| Maternal age in the first pregnancy, years | 27.93 + 4.83 | 28.36 + 4.31 | 0.015 |
| Ethnicity |  |  | 0.072 |
| Chinese | 317 (38.1) | 2600 (41.5) |  |
| Malay | 289 (34.8) | 1902 (30.4) |  |
| Indian | 83 (10.0) | 666 (10.6) |  |
| Others | 142 (17.1) | 1096 (17.5) |  |
| BMI at 12-week gestation in the first pregnancy, kg/m^2^ | 23.84 + 4.74 | 23.76 + 4.97 | 0.794 |
| BMI categories at 12-week gestation in the first pregnancy |  |  | 0.464 |
| Underweight (<18.5 kg/m^2^) | 28 (10.6) | 585 (9.3) |  |
| Normal weight (18.5-22.9 kg/m^2^) | 102 (38.4) | 2719 (43.4) |  |
| Overweight (23-27.4 kg/m^2^) | 81 (30.6) | 1785 (28.5) |  |
| Obesity (≥27.5kg/m^2^) | 54 (20.4) | 1175 (18.8) |  |
| BMI at 12-week gestation in the second pregnancy, kg/m^2^ | 24.55 + 4.98 | 24.92 + 5.40 | 0.138 |
| BMI categories at 12-week gestation in the second pregnancy |  |  | 0.730 |
| Underweight (<18.5 kg/m^2^) | 32 (6.3) | 416 (6.6) |  |
| Normal weight (18.5-22.9 kg/m^2^) | 189 (37.3) | 2262 (36.1) |  |
| Overweight (23-27.4 kg/m^2^) | 166 (32.7) | 1972 (31.5) |  |
| Obesity (≥27.5kg/m^2^) | 120 (23.7) | 1614 (25.8) |  |

Data are presented as number (percentage) for categorical variables and as mean + standard deviation for continuous variables. Sample size of excluded participants does not always add to n=831 due to missing data. BMI, body mass index.

^a^Based on Pearson chi-squared test for categorical variables and independent t-test for continuous variables.

**Supplementary Table S2. Comparisons of interpregnancy BMI change status and BMI categories in the second pregnancy by BMI categories in the first pregnancy based on World Health Organization conventional cut-offs (n=6264).**

|  | **BMI categories at 12-week gestation in the first pregnancy (kg/m^2^)** | | | |
| --- | --- | --- | --- | --- |
|  | <18.5 | 18.5 – 24.9 | 25 – 29.9 | ≥30 |
| **BMI change status between first and second pregnancy** |  |  |  |  |
| Stable -1 to <1 kg/m^2^ | 319 (54.5) | 1555 (41.6) | 417 (33.7) | 257 (36.4) |
| Loss >1 kg/m^2^ | 17 (2.9) | 354 (9.5) | 163 (13.2) | 109 (15.4) |
| Moderate gain 1 to <3 kg/m^2^ | 197 (33.7) | 1373 (36.8) | 385 (31.1) | 191 (27.0) |
| Excess gain ≥3 kg/m^2^ | 52 (8.9) | 454 (12.2) | 271 (21.9) | 150 (21.2) |
|  |  |  |  |  |
| **BMI categories at 12-week gestation in the second pregnancy** |  |  |  |  |
| <18.5 kg/m^2^ | 312 (53.3) | 104 (2.8) | 0 | 0 |
| 18.5 – 24.9 kg/m^2^ | 268 (45.8) | 2876 (77.0) | 98 (7.9) | 0 |
| 25 – 29.9 kg/m^2^ | 5 (0.9) | 737 (19.7) | 824 (66.7) | 45 (6.4) |
| ≥30 kg/m^2^ | 0 | 19 (0.5) | 314 (25.4) | 662 (93.6) |

Data are presented as number (percentage). BMI, body mass index.

**Supplementary Table S3. Association between crude interpregnancy BMI change measured at ≤12 weeks gestation and outcomes of second pregnancy (n=3755).**

|  | | **BMI change measured at ≤12 weeks gestation between first two pregnancies** | | | | |
| --- | --- | --- | --- | --- | --- | --- |
|  | | **Loss** |  | **Moderate gain** |  | **Excess gain** |
|  | | (>1 kg/m^2^) |  | (1 to <3 kg/m^2^) |  | (≥3 kg/m^2^) |
| **Outcomes of second pregnancy** | | RR (95% CI) |  | RR (95% CI) |  | RR (95% CI) |
| Offspring birth weight | |  |  |  |  |  |
|  | Low <2.5 kg (vs. Normal 2.5 to <4 kg) | 1.58 (1.05, 2.39) |  | 1.07 (0.80, 1.43) |  | 1.16 (0.78, 1.72) |
|  | High ≥4 kg (vs. Normal 2.5 to <4 kg) | 0.99 (0.44, 2.22) |  | 1.07 (0.60, 1.91) |  | 1.60 (0.83, 3.08) |
| Offspring birth size | |  |  |  |  |  |
|  | SGA <10 percentile (vs. AGA 10-90 percentile) | 1.56 (1.09, 2.25) |  | 0.95 (0.75, 1.22) |  | 0.83 (0.57, 1.20) |
|  | LGA >90 percentile (vs. AGA 10-90 percentile) | 1.02 (0.78, 1.33) |  | 1.23 (1.06, 1.44) |  | 1.47 (1.22, 1.76) |
| Preterm delivery <37 weeks | |  |  |  |  |  |
|  | Yes (vs. No) | 1.05 (0.67, 1.63) |  | 1.02 (0.76, 1.37) |  | 1.13 (0.77, 1.64) |
| Gestational diabetes | |  |  |  |  |  |
|  | Yes (vs. No) | 0.69 (0.47, 1.02) |  | 1.33 (1.07, 1.64) |  | 1.67 (1.31, 2.14) |
| Mode of delivery | |  |  |  |  |  |
|  | Elective caesarean (vs. vaginal delivery) | 0.98 (0.82, 1.16) |  | 1.03 (0.95, 1.12) |  | 1.16 (1.02, 1.31) |
|  | Emergency caesarean (vs. vaginal delivery) | 1.08 (0.82,1.43) |  | 1.22 (1.04, 1.43) |  | 1.14 (0.94, 1.40) |

Risk ratios are adjusted for maternal age and BMI at ≤12 weeks gestation in the first pregnancy, ethnicity, interpregnancy interval and respective pregnancy outcomes in the first pregnancy. BMI stable (-1 to <1 kg/m^2^) serves as the reference group. BMI, body mass index; RR, risk ratio; CI, confidence interval; AGA, appropriate for gestational age; SGA, small-for-gestational-age; LGA, large-for-gestational-age.

**Supplementary Figure S1**. Box and whisker plot showing the median and interquartile range of interpregnancy BMI loss by BMI categories of women at 12-week gestation in the first pregnancy. BMI categories were classified based on the cut-offs for Asian populations [11]. BMI, body mass index.
